# Development of a Droplet Digital PCR Assay Targeting the Internal Transcribed Spacer Gene for Rapid Detection of *Talaromyces marneffei* in AIDS Patients

**DOI:** 10.3390/pathogens14080817

**Published:** 2025-08-18

**Authors:** Yingyin Yang, Qinzhi Zhang, Pengle Guo, Meijun Chen, Yeyang Zhang, Weiping Cai, Xiaoping Tang, Linghua Li

**Affiliations:** 1 Infectious Disease Center, Guangzhou Eighth People’s Hospital, Guangzhou Medical University, Guangzhou 510440, Chinagz8hgpl@126.com (P.G.); gz8hcwp@126.com (W.C.); 2 Guangzhou Medical Research Institute of Infectious Diseases, Guangzhou 510440, China; 3 Department of Clinical Laboratory, Guangzhou Eighth People’s Hospital, Guangzhou Medical Diseases University, Guangzhou 510440, China; 4 Institute of Infectious Disease, Guangzhou Eighth People’s Hospital, Guangzhou Medical University, Guangzhou 510440, China; 5 Guangzhou Key Laboratory of Clinical Pathogen Research for Infectious Diseases, Guangzhou 510440, China

**Keywords:** *Talaromyces marneffei*, droplet digital PCR, detect, AIDS

## Abstract

Talaromycosis caused by *Talaromyces marneffei* is a life-threatening mycosis in patients with acquired immunodeficiency syndrome (AIDS). The gold-standard diagnostic method relies on time-consuming cultures, which delay treatment and increase mortality. In this study, we developed a rapid and sensitive droplet digital PCR (ddPCR) assay targeting the internal transcribed spacer (ITS) gene for detecting *T. marneffei* and compared its performance with blood culture and quantitative PCR (qPCR) assays. The ddPCR assay had a detection limit of one copy/reaction, making it 10-fold more sensitive than qPCR. It demonstrated 100% specificity for *T. marneffei*, with no cross-reactivity to 15 other fungal pathogens, six bacterial pathogens, and plasma from 119 AIDS patients without talaromycosis. In 119 AIDS patients with talaromycosis, ddPCR exhibited better overall sensitivity (92.44%) than blood culture (86.55%) and qPCR (87.29%). The sensitivity of ddPCR was 97.8% (89/91) and 75% (21/28) in plasma collected before and after antifungal therapy, respectively. Moreover, fungal load measured by ddPCR negatively correlated with the time to blood culture positivity. Fungal loads in patients receiving antifungal therapy were significantly lower than those in untreated patients. These findings indicate that ddPCR facilitates rapid diagnosis of *T. marneffei* infection in AIDS patients and can assist clinicians in evaluating treatment efficacy by quantifying fungal load.

## 1. Introduction

*Talaromyces marneffei* is a thermally dimorphic fungus endemic to Southeast Asia and southern China, and bamboo rats (*Rhizomys* spp.) serve as its natural reservoir [1]. It primarily causes fatal talaromycosis in immunocompromised individuals, particularly in people living with human immunodeficiency virus (HIV) who have a CD4^+^ T-cell count below 50 cells/μL [2]. The incidence of talaromycosis has increased since the emergence of the HIV pandemic. By mid-2022, more than 288,000 talaromycosis cases had been reported across 34 countries, accounting for a pooled prevalence of 3.6% among HIV-infected individuals [3]. In China, approximately 99% of cases were reported in the southern regions, with 43% and 41% in Guangxi and Guangdong, respectively. Of these, 88% occurred in HIV-infected individuals [4]. In endemic areas, talaromycosis is the third most common opportunistic infection (OI) in patients with acquired immunodeficiency syndrome (AIDS). An increasing number of cases have also been reported among non-HIV-infected patients, including those with primary immunodeficiency due to interferon-γ autoantibodies, autoimmune diseases, malignancy, or organ transplantations [5]. Currently, amphotericin B followed by itraconazole is the main treatment strategy for talaromycosis, whereas voriconazole serves as an alternative for those intolerant of amphotericin B. The mortality rate in HIV-infected patients is up to 30% even with antifungal therapy [6]. Despite the high morbidity and mortality, talaromycosis has received limited global attention to its diagnosis and treatment, prompting a global call in 2021 for its recognition as a neglected tropical disease [1] and leading to the inclusion of *T. marneffei* on the World Health Organization Fungal Priority Pathogen List in 2022 [7].

Common clinical manifestations include fever, skin lesions, and hepatosplenomegaly, which are not specific or reliable for diagnosis [6]. The gold standard for diagnosing talaromycosis is the isolation of *T. marneffei* in culture from blood or other clinical specimens; however, it can take up to 5–14 days, leading to delayed diagnosis and initiation of antifungal therapy [8]. The development of nonculture-based assays for rapid diagnosing talaromycosis is urgently required to improve treatment outcomes.

In recent years, quantitative polymerase chain reaction (qPCR) has been applied as a rapid, sensitive, and specific tool for detecting *T. marneffei* in clinical samples, including whole blood [9], plasma [10], and serum [11]. This method can quantify the fungal load based on a standard curve, which helps to evaluate disease progression and treatment efficacy. However, the drawbacks of qPCR should be considered, including suboptimal sensitivity in samples with low pathogen load, susceptibility to PCR inhibitors, and the need for a standard curve to quantify the results [12]. Standard curve production is strongly influenced by both lab-to-lab and day-to-day errors. Consequently, there is an emerging need for a more accurate tool to detect *T. marneffei* and quantify its level.

Droplet digital PCR (ddPCR), an emerging PCR technology, is a potential alternative to qPCR for microorganism detection. It separates target nucleic acids into tens of thousands of independent reaction droplets for amplification. Each droplet is categorized as positive or negative based on the presence or absence of the targets, allowing for absolute quantification of the targets using Poisson statistics without relying on a standard curve [13]. Compared with qPCR, ddPCR is more tolerant to PCR inhibitors because its partitioning process dilutes inhibitors across thousands of droplets, reducing their concentration in each droplet and minimizing their impact on amplification. Therefore, ddPCR has greater accuracy and sensitivity and is more suitable for detecting samples with a low pathogen load [14]. ddPCR has been used for the detection and quantification of several pathogens, such as HIV [15], *Candida* spp. [16], and *Mycobacterium tuberculosis* [17]. To our knowledge, ddPCR has not yet been studied for the detection of *T. marneffei*.

In this study, we developed a ddPCR method for highly sensitive and specific detection of *T. marneffei* in plasma from AIDS patients, and we performed a comprehensive comparison among ddPCR, qPCR and blood culture assays.

## 2. Materials and Methods

### 2.1. Study Population

**Figure 1:**
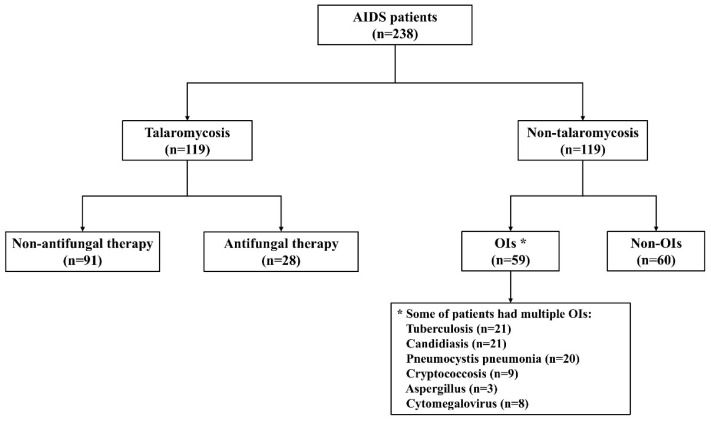
Study population used to assess the diagnostic performance of ddPCR.

This retrospective case–control study was conducted at Guangzhou Eighth People’s Hospital, Guangzhou Medical University, between June 2021 and December 2023. The adequate sample size was calculated using the PASS 11 software. We considered an area under the curve of >0.7 as the minimal standard, with α = 0.05, β of 0.10, and a ratio of the sample size of 1.0. A minimum sample size of 115 talaromycosis patients and 115 non-talaromycosis patients was needed. The study included a total of 238 participants (Figure 1). The inclusion criteria were as follows: (1) confirmed HIV infection by HIV antibody testing or HIV nucleic acid detection, (2) age ≥ 18 years, (3) blood culture performed within 3 days of admission, and (4) plasma samples collected within 3 days of admission. The exclusion criteria were the following: (1) pregnant or lactating women. According to the guidelines for the prevention and treatment of opportunistic infections [18], talaromycosis was diagnosed based on culture and/or histopathological evidence of *T. marneffei* from blood or other clinical samples. *T. marneffei* was isolated from positive cultures using standard culture techniques, and identification was based on morphological and microscopic examination of the colonies, as previously described [19]. Non-talaromycosis controls were defined as individuals with negative *T. marneffei* culture and/or histopathological results in blood or other clinical samples.

### 2.2. Strains

The following clinically related fungi were used in this study: *T. marneffei* ATCC 18224, *Penicillium citrinum* ATCC 10499, *C. albicans* ATCC 14053, *Cryptococcus neoformans* ATCC 14116, *Aspergillus flavus* ATCC 11489, *A. fumigatus* ATCC 46645, *A. terreus* ATCC 20542, *Fusarium oxysporum* ATCC 7601, *F. solani* ATCC 24388, as well as clinical isolates *T. diversiformis*, *T. purpureogenus*, *P. pimiteouiense*, *C. tropicalis*, *C. glabrata*, *F. proliferatum,* and *Histoplasma capsulatum*. Six bacterial pathogens were also used in this study, namely, *Salmonella enteritidi* ATCC 13076, *Pseudomonas aeruginosa* ATCC 27853, *Staphylococcus aureus* ATCC 29213, *Klebsiella pneumoniae* ATCC 700603, *Acinetobacter baumannii* ATCC BAA-747, and *M. tuberculosis* ATCC 25177. The bacteria or yeast forms of fungus were cultured on Luria–Bertani or Sabouraud–glucose plates at 37 °C for 2 to 7 days, respectively. The mold forms of fungus were cultured on a Sabouraud–glucose plate at 25 °C for 4 to 7 days. All experiments were performed in a biosafety level 2 laboratory.

### 2.3. DNA Extraction

Total DNA from pure culture or 400 μL plasma samples was extracted using QIAamp UCP Pathogen Mini Kit (QIAGEN, Hilden, Germany) with an elution volume of 50 μL. The DNA samples were stored at −80 °C until use.

### 2.4. ddPCR Assay

The internal transcribed spacer (ITS) sequences of *T. marneffei* strains from the NCBI database were aligned, and primers and a probe (Table S1) were designed to be highly specific for the conserved region. The ddPCR assay was performed using a TargetingOne ddPCR system according to the manufacturer’s instructions. A total of 30 μL of PCR mixture prepared for droplet generation contained 7.5 μL of 4 × PCR Mix (Targeting One, Singapore), 500 nM of each primer, 250 nM of probe, 5 μL of DNA template, and nuclease-free water. After droplet generation, PCR reaction was conducted using a Bio-Rad PTC 200 thermal cycler (Bio-Rad Laboratories, Hercules, CA, USA) according to the following conditions: 94 °C for 2 min, 40 cycles of 94 °C for 30 s, and 59 °C for 1 min. Finally, fluorescence signals from the droplets were measured using a Droplet Reader (Targeting One).

### 2.5. qPCR Assay

The qPCR was performed using a Bio-Rad CFX96 system. The PCR mixture was composed of 10 μL of TaqMan Fast Advanced Master Mix (Thermo Fisher Scientific, Waltham, MA, USA), 0.5 μL each of primers and probe (10 μM) used for ddPCR, 3.5 μL of sterile distilled water, and 5 μL of DNA template. The PCR condition was as follows: 95 °C for 20 s, 40 cycles of 95 °C for 3 s, and 59 °C for 30 s. The result was considered positive when the cycle threshold (Ct) value was ≤40 and negative when the Ct value was >40.

### 2.6. Assessment of Sensitivity and Specificity

The ITS gene from *T. marneffei* (accession no. L37406.1) was cloned into pUC57 plasmid and transformed into *Escherichia coli* DH5a. Successful transformants were selected through overnight culture in a Luria–Bertani plate supplemented with ampicillin. Finally, the pUC57-ITS recombinant plasmid was extracted using a Plasmid Mini Kit (Omega, Biel/Bienne, Switzerland). Ten-fold serial dilutions of the pUC57-ITS plasmid (10^6^–10^0^ copies/reaction) were used to examine the sensitivity of qPCR and ddPCR. Sensitivity was also evaluated using human plasma containing tenfold serial dilutions from 10^6^ to 10^1^ cells/mL of *T. marneffei*, and 400 μL of plasma of each dilution was used for DNA extraction. The specificity of qPCR and ddPCR for *T. marneffei* detection was evaluated using DNA from common clinically related pathogens.

### 2.7. Intra- and Inter-Assay Variability

Ten-fold dilutions of pUC57-ITS plasmids (10^6^–10^0^ copies/reaction) were examined in triplicate in one experiment, and the intra-assay coefficients of variation (CVs) were calculated. Three independent experiments were performed with these recombinant plasmid dilutions, and then the inter-assay CV was calculated.

### 2.8. Statistical Analysis

All data analyses were performed using SPSS 25.0 and GraphPad Prism 8.4.0. Continuous variables are expressed as mean and standard deviation (SD) or as the median and interquartile range (IQR). Comparison of continuous variables between the two groups was performed using the independent-sample *t*-test or Mann–Whitney U test, as appropriate. The chi-squared test was used to analyze categorical variables, which were expressed as frequencies (percentages). Pearson’s correlation test was used to analyze correlations between different groups. The sensitivity, specificity, positive predictive value (PPV), negative predictive value (NPV), and kappa value were calculated to evaluate the diagnostic performance of ddPCR and other diagnostic methods. Statistical significance was considered with *p*-value ≤ 0.05.

## 3. Results

### 3.1. Analytical Specificity and Sensitivity of ddPCR

**Figure 2:**
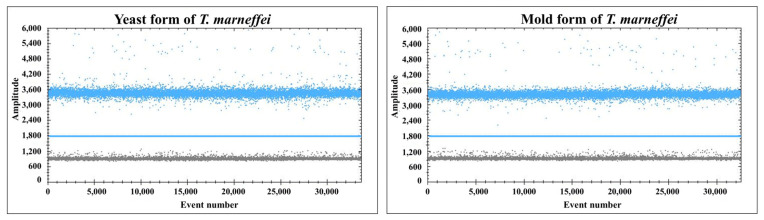
ddPCR assay for *T. marneffei*. The blue line represents the threshold, and the positive and negative droplets are shown in blue and black, respectively.

In the ddPCR assay, the yeast and mold forms of *T. marneffei* produced positive droplets (Figure 2), whereas no positive droplets were observed in the other six bacterial pathogens and 15 fungal pathogens, such as *Talaromyces* spp., *Penicillium* spp., *Aspergillus* spp., *Candida* spp., *Fusarium* spp., and *H. capsulatum*. Consistently, in the qPCR assay, a positive Ct value was detected only for *T. marneffei* among the tested strains (Table S2). Thus, the qPCR and ddPCR assays were highly specific for *T. marneffei*, with 100% specificity.

**Figure 3:**
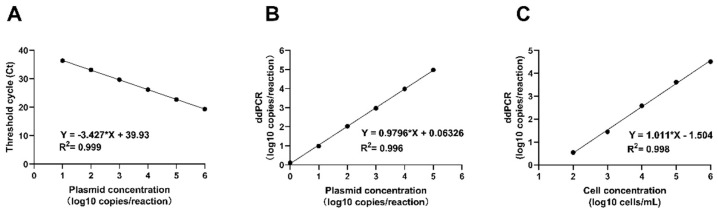
Assessment of the dynamic range of qPCR and ddPCR. The linearity range of (**A**) qPCR and (**B**) ddPCR for the quantification of *T. marneffei* pUC57-ITS plasmids. (**C**) The linearity range of ddPCR for the quantification of *T. marneffei* yeast cells.

Ten-fold dilutions of the pUC57-ITS plasmids were used to construct standard curves for qPCR and ddPCR. Both methods exhibit a good linear range (Figure 3). The detection limit was 10 copies/reaction (or 250 copies/mL) in qPCR, whereas ddPCR was one copy/reaction (or 25 copies/mL), indicating that ddPCR was more sensitive for *T. marneffei* detection. However, when the target exceeded 10^5^ copies/reaction, ddPCR could not be accurately quantified as the number of positive droplets reached saturation. Additionally, good linearity between the concentration of *T. marneffei* yeast and the value measured by ddPCR was observed. The value measured at the detection limit of 100 cells/mL was 3.5 copies/reaction (or 87.5 copies/mL), indicating DNA loss during extraction.

### 3.2. Intra- and Inter-Assay Variability of ddPCR

To evaluate the intra- and inter-assay variability of ddPCR, various concentrations of the pUC57-ITS plasmids were tested in triplicate for ddPCR. The intra-assay CV of different gradients ranged from 0.016 to 0.078, and the inter-assay CV ranged between 0.021 and 0.204 (Table S3), suggesting that the ddPCR assay had good repeatability and reproducibility.

### 3.3. Characteristics of the Study Population at Baseline

Among the 119 patients with AIDS and culture-proven talaromycosis included in this study, 103 were positive for *T. marneffei* in blood culture, and 16 were negative in blood culture but positive in bone marrow or bronchoalveolar lavage fluid culture. A total of 119 AIDS patients without talaromycosis were included as controls, including 54 patients without OIs and 45 with at least one other OI (21 with tuberculosis, 21 with candidiasis, 20 with pneumocystis pneumonia, 9 with cryptococcosis, 3 with aspergillosis, and 8 with cytomegalovirus infection). The talaromycosis group had a significantly higher plasma HIV RNA load (5.70 vs. 4.58, *p* < 0.001), lower CD4^+^ cell count (12 vs. 109, *p* < 0.001), and lower CD4^+^/CD8^+^ ratio (0.06 vs. 0.19, *p* < 0.001) than the non-talaromycosis group, as previously reported [19]. Additionally, there were no significant differences observed in age and sex between the two groups (*p* > 0.05). The baseline clinical characteristics of the study population are listed in Table 1.

### 3.4. Diagnostic Performance of ddPCR

For the diagnosis of talaromycosis in AIDS patients, the sensitivity, specificity, PPV, NPV, and kappa value of blood culture were 86.55%, 100%, 100%, 88.15%, and 0.866, respectively; qPCR demonstrated similar detection efficiencies, with a sensitivity, specificity, PPV, NPV, and kappa value of 87.39%, 100%, 100%, 88.81%, and 0.874, respectively; ddPCR showed the highest detection efficiency, with a sensitivity, specificity, PPV, NPV, and kappa value of 92.44%, 100%, 100%, 92.97%, and 0.924, respectively (Table 2). However, six talaromycosis cases were negative according to ddPCR but positive according to blood culture.

At the time of blood sample collection, patients in the non-talaromycosis group with other fungal infections had not received antifungal therapy. Among talaromycosis patients, 91 (76.47%) had not received antifungal therapy, whereas 28 (23.53%) had received antifungal therapy prior to admission (Table 3). Of those who had received antifungal therapy, 96.43% (27/28) were treated with azole drugs, and 3.57% (1/28) were treated with echinocandins. The median duration of antifungal therapy was 7 days (IQR 3–10.5). In the non-antifungal therapy group, ddPCR had a higher positive rate (89/91, 97.80%) than for qPCR (83/91, 91.21%, *p* = 0.051) or blood culture (79/91, 86.81%, *p* = 0.005). In the antifungal therapy group, the positivity rates were lower for qPCR and ddPCR (21/28, 75.00%) than for blood culture (24/28, 85.71%, *p* = 0.313), though the differences were not statistically significant (*p* > 0.05). These results indicate that ddPCR is advantageous in detecting talaromycosis in patients not receiving antifungal therapy.

### 3.5. Fungal Load Analysis in Clinical Samples

**Figure 4:**
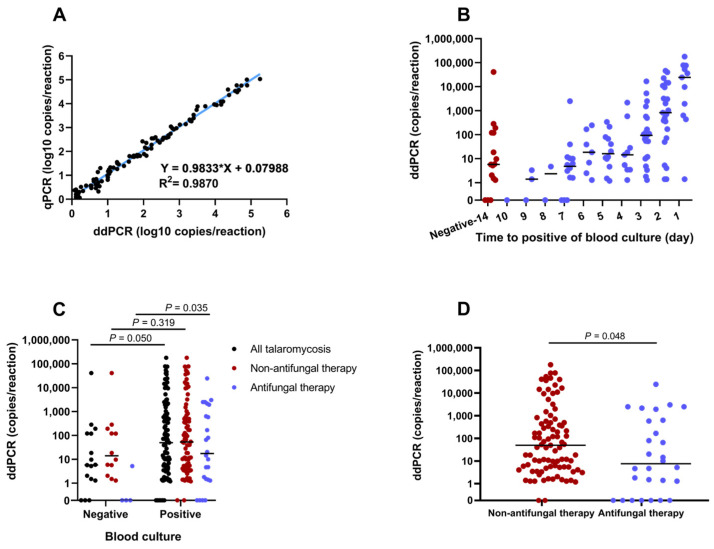
Performance of ddPCR in clinical samples from patients with talaromycosis. (**A**) Correlation between the results of qPCR and ddPCR in positive samples. The copy numbers of qPCR were calculated with the standard curve and Ct values. (**B**) Relationship between fungal load and time to positive blood culture. Fungal load of *T. marneffei* was measured with ddPCR and presented as copies/reaction. (**C**) Comparison of fungal load in different blood culture results. (**D**) Effect of antifungal therapy on the fungal load of *T. marneffei*. The black lines in (**B**–**D**) represented the median value. Statistical significance was considered as *p* ≤ 0.05.

Samples with positive results for ddPCR and qPCR were used for the linear regression analysis. A good correlation between the copy number measured using ddPCR and the copy number calculated using qPCR was observed, with a correlation coefficient of 0.9935 (Figure 4A). We used the ddPCR results for further analysis, since ddPCR could quantify the target without relying on a standard curve.

In all patients with talaromycosis, the median time for detecting the growth signal in blood culture was 4 (IQR 2–7) days, and the measured copy number of ddPCR increased with a decrease in the time to positivity of blood culture (Figure 4B). Regardless of antifungal therapy, patients with talaromycosis confirmed by blood culture had higher median copies in plasma than patients with negative blood culture results (Figure 4C). These results indicated that the degree of fungal load in the plasma was related to the blood culture outcome. Moreover, the median copy number was significantly higher in the non-antifungal therapy group than in the antifungal therapy group (50.1 vs. 7.62, *p* = 0.048; Figure 4D), indicating that antifungal therapy could influence the fungal load.

## 4. Discussion

Timely diagnosis of *T. marneffei* can control the source of infection and enable the implementation of targeted treatment measures. Detection with molecular methods, such as PCR, could significantly reduce the diagnosis time due to the long period required for fungal growth in cultures. qPCR, a well-developed method for the detection of pathogenic fungi, has shown great potential for diagnosing *T. marneffei* infection. The diagnostic sensitivity of qPCR in patients with talaromycosis ranges from 60 to 86%, and with a specificity of 100% [9,10,11]. As a new-generation technology, ddPCR has exhibited greater sensitivity than qPCR in many studies [16,20,21], which may improve the sensitivity of detection of *T. marneffei*.

In this study, we developed a ddPCR assay to detect *T. marneffei*. ITS, a multi-copy gene in the fungal genome, was selected as the target gene owing to its higher sensitivity compared to the Mp1 gene in previous qPCR assays for diagnosing *T. marneffei* infection (86.11% vs. 70.4%) [8,9]. The designed primers and probe were 100% specific to *T. marneffei* with no cross-reactivity to other common clinically pathogenic fungi and bacteria, as well as to plasma samples from AIDS patients without talaromycosis. Furthermore, ddPCR exhibited a superior analytical sensitivity of one copy/reaction, compared to 10 copies/reaction for our qPCR assay and another qPCR assay using the same target gene [11]. The detection limit of ddPCR for *T. marneffei* cells spiked in plasma was only 100 cells/mL, which was consistent with the result of another qPCR assay using lysozyme to break down the cell wall [10]. To overcome the challenge of the fungal cell wall hindering DNA extraction, it is necessary to increase the detection limit of ddPCR to 1–10 cells/mL by improving the extraction method in future studies.

In patients with talaromycosis, qPCR and ddPCR results were mostly consistent. However, there were six cases that were negative according to qPCR but positive according to ddPCR, with copy numbers ranging from 1.3 to 5.7. Thus, ddPCR had higher diagnostic sensitivity than qPCR (92.44% vs. 87.99%) and was more suitable for detecting samples with a low copy number, mainly because ddPCR was less prone to PCR inhibitors [12]. The diagnostic sensitivity of our ddPCR assay was also higher than other qPCR assays (86.11%, 31/36; 62%, 31/50; 60%, 12/20) [9,10,11]. Additionally, ddPCR had a great advantage over qPCR in quantifying copy numbers without requiring a calibration curve. Although the upper detection limit of ddPCR (105 copies/reaction) was 10-fold lower than that of qPCR, we observed that the median copies for ddPCR detection among talaromycosis patients was 39.2 copies/reaction (IQR 39.2–568.6), which was considerably lower than the upper detection limit.

Compared with the 5–10 mL volume of blood used in blood culture, our ddPCR assay detected more positive samples and demonstrated higher diagnostic sensitivity (92.44% vs. 86.55%) while requiring 400 μL of plasma, roughly equivalent to 1 mL of blood. Notably, six cases of talaromycosis were undetected by ddPCR but yielded positive blood cultures in the second week. This discrepancy likely reflects the blood culture’s ability to detect viable fungi at extremely low concentrations through growth amplification, whereas ddPCR may yield false-negative results when the fungal load falls below its detection limit. Increasing the blood volume for DNA extraction could improve ddPCR sensitivity, but it may reduce patient compliance and the convenience of blood collection [22]. Furthermore, the median time to identify *T. marneffei* using blood culture was 4 days, while ddPCR significantly shortened the reporting time to 4 h, enabling a rapid diagnosis.

It is well known that the pathogenic load in patients can decrease after appropriate therapy. Our study observed that the median fungal load in samples collected before antifungal therapy (50.1 copies/reaction) was significantly higher than that in samples collected after therapy (7.62 copies/reaction). Correspondingly, the diagnostic sensitivity of ddPCR was reduced from 97.8% in patients without therapy to 75% in patients receiving therapy, which was similar to the results of the qPCR assay for *T. marneffei* infections [10]. Another study also reported that ddPCR detected bloodstream infections with 100% sensitivity in patients without treatment, with a sensitivity of 71.43% in empirically treated patients [23]. Given that the sensitivity of blood culture was approximately 86% in both groups, the combination of ddPCR and blood culture tests may be more suitable for the timely diagnosis of *T. marneffei* infection in patients who have already received treatment.

This study had several limitations. First, as a retrospective case–control study, the selected study population may not fully reflect real-world clinical settings, potentially leading to an overestimation of diagnostic accuracy. Second, only plasma samples were available for ddPCR evaluation in this study. Whole blood may be a more suitable diagnostic specimen, since *T. marneffei* primarily resides in macrophages [24] and could be lost during plasma sample processing. Third, although ddPCR demonstrates high sensitivity for detecting *T. marneffei* DNA, it cannot differentiate between live and dead cells [25], which could result in false-positive results even after pathogen clearance. Fourth, compared with qPCR, ddPCR requires approximately 30 additional minutes for droplet processing and post-amplification analysis. It also requires specialized equipment, which increases operational complexity and costs.

In conclusion, our study established a ddPCR method for detecting *T. marneffei* in plasma samples, with an overall sensitivity of 92.44% and a specificity of 100%. It can be used as a valuable tool to rapidly diagnose *T. marneffei* infection in AIDS patients and to guide treatment decisions.

## Figures and Tables

**Table 1:** Characteristics of the study population at baseline.

Characteristics	Talaromycosis Group (n = 119)	Non-Talaromycosis Group (n = 119)	*p* Value
Age (years)	40 (31–51)	43 (33–54)	0.107
Male	105 (88.24)	99 (83.19)	0.267
Plasma HIV RNA, log10 IU/mL	5.70 (5.23–6.30)	4.58 (2.23–5.50)	<0.001
CD4^+^ cell (cells/uL)	12 (6.0–27.0)	109 (34–296)	<0.001
CD4^+^/CD8^+^ ratio	0.06 (0.03–0.14)	0.19 (0.07–0.40)	<0.001

**Table 2:** Comparison of the diagnostic performance of blood culture, qPCR, and ddPCR for talaromycosis in AIDS patients.

Test	Sensitivity	Specificity	PPV	NPV	Kappa
Blood culture	86.55% (103/119)	100% (119/119)	100% (103/103)	88.15% (119/135)	0.866
qPCR	87.39% (104/119)	100% (119/119)	100% (104/104)	88.81% (119/134)	0.874
ddPCR	92.44% (110/119)	100% (119/119)	100% (110/110)	92.97% (119/128)	0.924

**Table 3:** Comparison of diagnostic performance in talaromycosis patients with and without antifungal therapy.

Test Positive	Non-Antifungal Therapy (n = 91)	Antifungal Therapy (n = 28)	*p* Value
Blood culture	86.81% (79/91)	85.71% (24/28)	1.000
qPCR	91.21% (83/91)	75.00% (21/28)	0.045
ddPCR	97.80% (89/91)	75.00% (21/28)	0.001

## Data Availability

All relevant data are within the manuscript and its Supporting Information files.

## Supplementary Materials

The following supporting information can be downloaded at: https://www.mdpi.com/article/10.3390/pathogens14080817/s1, Table S1: Primers and probe used in this study; Table S2: Specificity of qPCR and ddPCR; Table S3: Intra- and inter-assay variability of ddPCR.
