# Supplementary material for: Development of a Droplet Digital PCR Assay Targeting the Internal Transcribed Spacer Gene for Rapid Detection of Talaromyces marneffei in AIDS Patients
Source: Pathogens. 2025 Aug 18;14(8):817. doi: 10.3390/pathogens14080817 (PMC12389560; doi:10.3390/pathogens14080817)
Supplement: Supplementary file 1 [file pathogens-14-00817-s001.zip › Table S1.pdf]

Table S1. Primers and probe used in this study.

Primers and probe	Nucleotide sequences
Forward primer	5’-CTGTCCGAGCGTCATTTC-3’
Reverse primer	5’-AGGTCAACCGTGGTAAATATG-3’
Probe	5’-FAM-CTTGTGTGTTGGGTGTGGTCCCTC-BHQ1-3’
