# Supplementary material for: Development of a Droplet Digital PCR Assay Targeting the Internal Transcribed Spacer Gene for Rapid Detection of Talaromyces marneffei in AIDS Patients
Source: Pathogens. 2025 Aug 18;14(8):817. doi: 10.3390/pathogens14080817 (PMC12389560; doi:10.3390/pathogens14080817)
Supplement: Supplementary file 1 [file pathogens-14-00817-s001.zip › Table S2.pdf]

Table S2. Specificity of qPCR and ddPCR.

DNA samples	qPCR	ddPCR
Yeast form of *T. marneffei*	Positive	Positive
Mould form of *T. marneffei*	Positive	Positive
T. diversiformis	Negative	Negative
T. purpureogenus	Negative	Negative
P. pimiteouiense	Negative	Negative
P. citrinum	Negative	Negative
C. albicans	Negative	Negative
C. tropicalis	Negative	Negative
C. glabrata	Negative	Negative
C. neoformans	Negative	Negative
A. flavus	Negative	Negative
A. fumigatus	Negative	Negative
A. terreus	Negative	Negative
H. capsulatum	Negative	Negative
F. oxysporum	Negative	Negative
F. solani	Negative	Negative
F. proliferatum	Negative	Negative
S. enteritidi	Negative	Negative
P. eruginosa	Negative	Negative
S. aureus	Negative	Negative
K. pneumoniae	Negative	Negative
A. baumannii	Negative	Negative
M. tuberculosis	Negative	Negative
