# Supplementary material for: Development of a Droplet Digital PCR Assay Targeting the Internal Transcribed Spacer Gene for Rapid Detection of Talaromyces marneffei in AIDS Patients
Source: Pathogens. 2025 Aug 18;14(8):817. doi: 10.3390/pathogens14080817 (PMC12389560; doi:10.3390/pathogens14080817)
Supplement: Supplementary file 1 [file pathogens-14-00817-s001.zip › Table S3.pdf]

Table S3. Intra- and inter-assay variability of ddPCR.

pUC57-ITS plasmid (copies/reaction)	Intraassay variation			Interassay variation		
	**Mean**	**SD**	**CV**	**Mean**	**SD**	**CV**
**10^5**	94742.40	4986.94	0.053	96022.47	6427.87	0.067
**104**	9319.77	535.12	0.057	9742.97	541.18	0.056
**10^3**	940.73	14.78	0.016	936.37	19.56	0.021
**10^2**	109.93	5.39	0.049	106.87	8.10	0.076
**10**	9.47	0.74	0.078	9.73	1.50	0.155
**1**	1.25	0.07	0.057	1.30	0.26	0.204
